# Birth Weight, Gestational Age, and Risk of Pediatric-Onset MASLD

**DOI:** 10.1001/jamanetworkopen.2024.32420

**Published:** 2024-09-10

**Authors:** Fahim Ebrahimi, Jialu Yao, Hannes Hagström, Olof Stephansson, Jiangwei Sun, David Bergman, Jonas Söderling, Jonas F. Ludvigsson

**Affiliations:** 1 Department of Medical Epidemiology and Biostatistics, Karolinska Institutet, Stockholm, Sweden; 2 Department of Gastroenterology and Hepatology, Clarunis University Center for Gastrointestinal and Liver Diseases, Basel, Switzerland; 3 Division of Hepatology, Department of Upper GI, Karolinska University Hospital, Stockholm, Sweden; 4 Department of Medicine, Huddinge, Karolinska Institutet, Stockholm, Sweden; 5 Department of Women’s Health, Karolinska University Hospital, Stockholm, Sweden; 6 Division of Clinical Epidemiology, Department of Medicine, Solna, Karolinska Institutet, Stockholm, Sweden; 7 Department of Pediatrics, Örebro University Hospital, Örebro, Sweden; 8 Department of Medicine, Columbia University College of Physicians and Surgeons, New York, New York

## Abstract

**Importance:**

Metabolic dysfunction–associated steatotic liver disease (MASLD) has become the most common chronic liver disease worldwide and is increasingly being diagnosed at younger ages, affecting more than one-third of young people with obesity.

**Objective:**

To evaluate associations between perinatal conditions and risk of MASLD and associated progressive liver disease.

**Design, Setting, and Participants:**

This nationwide, population-based case-control study included all biopsy-confirmed cases of MASLD in Sweden. Individuals aged 25 years or younger (hereafter, young individuals) with biopsy-proven MASLD between January 1, 1992, and December 31, 2016, were matched to up to 5 general population control individuals. Granular data on maternal and perinatal characteristics were retrieved from the Swedish Medical Birth Register. Data were analyzed from June 2023 to June 2024.

**Exposures:**

Birth weight (low [<2500 g], reference [2500 to <4000 g], or high [≥4000 g]), gestational age (GA), and birth weight for GA (small for GA [SGA; <10th percentile], appropriate for GA [10th-90th percentile], or large for GA [LGA; >90th percentile]), compared between patients and matched controls.

**Main Outcomes and Measures:**

The main outcome was odds of biopsy-proven MASLD and MASLD-associated progressive liver disease (ie, liver fibrosis or cirrhosis) according to birth weight, GA, and birth weight for GA, adjusted for matching factors.

**Results:**

In total, 165 young individuals with biopsy-proven MASLD (median age at diagnosis: 12.0 years [IQR, 4.4-16.9 years]; 100 [60.6%] male) were matched with 717 controls. There was an association between low birth weight and future development of MASLD (adjusted odds ratio [AOR], 4.05; 95% CI, 1.85-8.88) but no association between high birth weight and odds of MASLD (AOR, 0.64; 95% CI, 0.38-1.08) compared with the reference birth weight. An association was seen for SGA (AOR, 3.36; 95% CI, 2.00-5.64) compared with appropriate size for GA (reference category) but not for LGA (AOR, 0.57; 95% CI, 0.27-1.20). Progressive liver disease was more common in individuals born with low birth weight (AOR, 6.03; 95% CI, 1.66-21.87) or SGA (AOR, 4.90; 95% CI, 2.15-11.14).

**Conclusions and Relevance:**

In this nationwide study of young individuals with biopsy-proven MASLD, low birth weight and SGA were associated with development of MASLD and progressive liver disease, suggesting a need for structured screening measures to diagnose these conditions early in high-risk individuals.

## Key Points

**Question:**

Are perinatal conditions associated with risk of development of metabolic dysfunction–associated steatotic liver disease (MASLD)?

**Findings:**

In this nationwide case-control study of 165 Swedish individuals aged 25 years or younger with biopsy-proven MASLD matched with 717 control individuals, associations were found between low birth weight and small for gestational age and higher risk of pediatric-onset MASLD and progressive liver disease.

**Meaning:**

The findings suggest that newborns with low birth weight or born small for gestational age have higher risk of developing pediatric-onset MASLD or progressive liver disease.

## Teaser

This case-control study investigates the association of birth weight and gestational age with risk of metabolic dysfunction–associated steatotic liver disease (MASLD) among children, adolescents, and young adults in Sweden.

## Introduction

Metabolic dysfunction–associated steatotic liver disease (MASLD; formerly known as nonalcoholic fatty liver disease) has become the most common chronic liver disease worldwide^1,2^ and is increasingly being diagnosed at younger ages, affecting more than one-third of young people with obesity.^3,4^ There is a high degree of interindividual heterogeneity in the risk of developing MASLD-associated progressive liver disease (ie, liver fibrosis or cirrhosis) that cannot be explained by the degree of obesity or genetic risks alone.^5^ While MASLD remains stable for decades in most affected individuals, only a few experience progressive liver disease with the development of the inflammatory subtype metabolic dysfunction–associated steatohepatitis (MASH), which can further progress to various stages of liver fibrosis and ultimately cirrhosis.^6^ Compared with MASLD in adults, pediatric-onset MASLD appears to have an even more aggressive natural history, with a higher incidence among young individuals already having histologic evidence of MASH and at stages of advanced fibrosis^7^ or even cirrhosis^8^ at the time of diagnosis.

Therefore, it has been hypothesized that MASLD and particularly MASLD-associated progressive liver disease may have developmental origins in early life.^9^ Several studies have provided the basis for the Developmental Origins of Health and Disease hypothesis,^10,11,12^ suggesting that both intrauterine states of undernutrition^13^ and overnutrition^14^ induce epigenetic adaptations that significantly influence an individual’s metabolism and risk of developing cardiometabolic disease in later life.^15^ A key objective parameter to measure the complex interactions between fetal adaptations and epigenetic programming is birth weight.^16^ Several studies have shown a U-shaped association between birth weight and the development of obesity,^17^ metabolic syndrome,^18^ and cardiovascular disease,^19^ but the evidence for MASLD is less clear. A previous systematic review stated that adequately powered studies based on comprehensive liver histologic findings and granular data on maternal and perinatal characteristics are urgently needed.^20^ Herein, we aimed to fill the identified knowledge gap by conducting a nationwide, matched case-control study including all individuals aged 25 years or younger in Sweden with biopsy-proven MASLD to investigate associations of birth weight, gestational age (GA), and birth weight for GA with risks of MASLD and MASLD-associated progressive liver disease.

## Methods

We conducted a population-based, matched case-control study using the nationwide Epidemiology Strengthened by Histopathology Reports in Sweden (ESPRESSO) cohort, which encompasses all liver histopathologic examination findings from all 28 pathology departments in Sweden between 1965 and 2017.^21^ This study was approved by the Ethics Review Board in Stockholm, Sweden. Informed consent was waived as the study was registry based.^22^ The study followed the Strengthening the Reporting of Observational Studies in Epidemiology (STROBE) reporting guideline.^23^

### Data Sources

With use of the unique Swedish personal identity number assigned to all residents at birth or at the time of legal residence,^24^ each individual in the ESPRESSO cohort was linked to validated nationwide registers^25,26,27,28^ that prospectively record detailed data on demographics, socioeconomics, and disease and comorbidity development. For maternal and perinatal data, we used data from the nationwide Swedish Medical Birth Register (MBR).^29^ In Sweden, every pregnant individual is offered routine structured antenatal visits at defined intervals, during which the medical history is retrieved, a clinical examination is carried out, and ultrasonography is performed. Information collected includes granular data on previous obstetric history (eg, number of live births, miscarriages), pregnancy-related complications (ie, gestational diabetes, preeclampsia), smoking status, body mass index (BMI; calculated as weight in kilograms divided by height in meters squared), medication use, and any maternal diseases. Furthermore, detailed data from obstetric and neonatal care at the time of birth, including mode of delivery, GA, birth weight, and infant diagnoses and complications at birth, are recorded.

### Study Population

For the nationwide case-control study, we included all children, adolescents, and young adults aged 25 years or younger with a biopsy-proven diagnosis of MASLD who were born in Sweden between January 1, 1992, and December 31, 2016, as relevant parameters such as maternal BMI were first recorded during this period. Individuals aged 10 years or younger were defined as children, 11 to 17 years as adolescents, and 18 to 25 years as young adults. We used a validated algorithm for the identification of histologically confirmed MASLD, which has shown a positive predictive value of 91.3%.^30^ In brief, we excluded any individual with any concomitant chronic liver disease on or before the index date, including alcohol use disorder or drug abuse, drug-induced liver injury, hepatitis B or C virus infection, HIV infection, autoimmune hepatitis, primary biliary cholangitis, primary sclerosing cholangitis, α_1_-antitrypsin deficiency, Wilson disease, or rare pediatric liver diseases that could cause steatosis (ie, inborn errors of metabolism), or prior use of steatogenic medications (detailed definitions are shown in eTable 1 in Supplement 1).

Individuals meeting the criteria for MASLD were then classified into 2 main groups according to liver histologic findings using Systematized Nomenclature of Medicine definitions for consistent nationwide histopathology reporting in Sweden^31^: (1) nonsevere MASLD, including simple steatosis or MASH without fibrosis, and (2) MASLD-associated progressive liver disease, including MASLD with noncirrhotic fibrosis or cirrhosis due to MASLD, commonly referred to as progressive liver disease. Each individual was then matched to up to 5 reference individuals from the general population (control individuals) without recorded MASLD on the basis of age at the index (diagnosis) date, sex, calendar year of the index date, and county of residence. Controls were derived from the Swedish Total Population Register,^25^ and identical exclusion criteria were applied to ensure that controls did not have a prior diagnosis of any other concomitant liver disease at or before baseline. For each individual, we further retrieved data from the Swedish National Patient Register on relevant comorbidities at the time of MASLD diagnosis or matching, including cardiovascular diseases, diabetes, hypertension, and dyslipidemia.

### Exposures

Using the personal identity number of included individuals, we identified mothers of patients with biopsy-proven MASLD and controls and retrieved detailed data on maternal and pregnancy-related parameters from the MBR. Gestational age at delivery was estimated by ultrasonography when available or otherwise by date of the last menstrual period and was subsequently categorized as preterm (<37 weeks), full term (37-41 weeks, used as the reference category), or postterm (≥42 weeks). Birth weight was categorized as low (<2500 g), reference (2500 to <4000 g), or high (≥4000 g). Birth weight for GA was defined according to the World Health Organization (WHO) definition^32^ as small for GA (SGA; <10th percentile), appropriate for GA (10th-90th percentile, used as the reference category), or large for GA (LGA; >90th percentile).

### Statistical Analysis

We performed conditional logistic regression analyses to evaluate the odds of MASLD according to birth weight, GA, and birth weight for GA. We estimated conditional and adjusted odds ratios (AORs) and their 95% CIs conditioned on matching factors (age, sex, calendar year, and county of residence) and further adjusted for known or suspected maternal factors that may affect the odds for developing MASLD in offspring: maternal age, maternal early-pregnancy BMI, maternal country of birth (Nordic, other), parity, parents’ highest level of education (≤9 years, 10-12 years, ≥13 years, or missing data), and smoking in early pregnancy (nonsmoker, smoking 1-9 or ≥10 cigarettes per day, or missing data) (definitions of covariates are presented in eTable 2 in Supplement 1). The association between birth weight and odds of MASLD was graphically depicted using a restricted cubic spline function for birth weight with knots at 1000 g, 2000 g, 3000 g, and 4000 g, and ORs were adjusted.

We conducted stratified analyses according to sex. Furthermore, we conducted several sensitivity analyses to test the robustness of our results. First, to explore whether any association between birth weight, GA, and birth weight for GA and MASLD might be influenced by pregnancy-related metabolic morbidities, we performed sensitivity analyses additionally adjusting for preeclampsia (uteroplacental insufficiency has been associated with a higher metabolic risk^33^) and gestational diabetes (offspring of mothers with gestational diabetes were found to have a higher risk of hepatic steatosis^34^), which have both been suspected to be part of the causal pathway of MASLD. Second, to address potential residual confounding related to suspected or undiagnosed inborn errors of metabolism, we excluded individuals younger than 2 years with liver biopsy confirmation of MASLD. Third, because diagnostic definitions have changed over time, we restricted analysis to individuals diagnosed with MASLD since 2004. Fourth, we identified all full siblings of individuals with MASLD and performed a sibling-controlled analysis, which enabled us to account for potential intrafamilial confounding due to genetic factors and early environmental exposures. Sibling-controlled analyses were conditioned on family, age, sex, and birth year and further for known or suspected maternal factors that may affect the odds of developing MASLD (maternal age, maternal early-pregnancy BMI, maternal country of birth, parity, parents’ highest level of education, and smoking in early pregnancy). Statistical analyses were conducted from June 2023 to June 2024 using SAS, version 9.4 (SAS Institute Inc) and Stata, version 17.0 (StataCorp LLC). Two-sided *P* < .05 was considered statistically significant.

## Results

### Patient Characteristics

In total, we identified 165 individuals in the ESPRESSO cohort with a diagnosis of MASLD confirmed by liver biopsy between January 1, 1992, and December 31, 2016, of whom 70 (42.4%) were children, 70 (42.4%) were adolescents, and 25 (15.2%) were young adults. They were matched to 717 controls (324 children [45.2%], 297 adolescents [41.4%], and 96 young adults [13.4%]) after the same exclusion criteria were applied (the flowchart of study inclusion is shown in the eFigure in Supplement 1). The characteristics of all individuals with MASLD and controls are summarized by age category in the Table and overall in eTable 3 in Supplement 1. The median age at diagnosis of individuals with MASLD was 12.0 years (IQR, 4.4-16.9 years); 65 (39.4%) were female, and 100 (60.6%) were male. According to histologic findings, 62 (37.6%) were diagnosed with simple steatosis, 27 (16.4%) with MASH without fibrosis, 71 (43.0%) with MASLD with noncirrhotic fibrosis, and 5 (3.0%) with cirrhosis due to MASLD. Despite their young age, at the time of liver biopsy, 9 individuals with MASLD (5.5%) had developed diabetes compared with 3 controls (0.4%). A significant proportion of young individuals with MASLD already had clinical evidence of cardiovascular disease (13 cases [7.9%], compared with 13 controls [1.8%]). The early-pregnancy BMI was higher in mothers whose offspring were later diagnosed with MASLD compared with mothers of controls (25.0 vs 23.1; *P* < .001).

**Table.** Characteristics of Children, Adolescents, and Young Adults With MASLD and Matched Control Individuals at Birth and the Index Date^a^

Characteristic	Children		Adolescents		Young adults	
	With MASLD (n = 70)	Controls (n = 324)	With MASLD (n = 70)	Controls (n = 297)	With MASLD (n = 25)	Controls (n = 96)
**At birth**						
Maternal age, y						
Median (IQR) [range]	29.5 (25.2-34.4) [17.8-40.5]	29.7 (25.9-33.5) [18.0-44.8]	29.2 (25.6-33.2) [18.3-42.8]	29.7 (26.0-32.8) [18.8-42.4]	27.4 (24.7-30.7) [20.0-40.5]	29.2 (26.0-32.9) [17.5-40.0]
≤24	16 (22.9)	55 (17.0)	17 (24.3)	57 (19.2)	8 (32.0)	16 (16.7)
25-29	19 (27.1)	117 (36.1)	20 (28.6)	99 (33.3)	8 (32.0)	37 (38.5)
30-34	20 (28.6)	89 (27.5)	19 (27.1)	96 (32.3)	6 (24.0)	29 (30.2)
≥35	15 (21.4)	63 (19.4)	14 (20.0)	45 (15.2)	3 (12.0)	14 (14.6)
Birth year						
1992-1999	34 (48.6)	156 (48.1)	43 (61.4)	183 (61.6)	25 (100)	96 (100)
2000-2010	34 (48.6)	159 (49.1)	27 (38.6)	114 (38.4)	0	0
2011-2016	2 (2.9)	9 (2.8)	0	0	0	0
Maternal country of birth						
Nordic country	51 (72.9)	273 (84.3)	48 (68.6)	260 (87.5)	21 (84.0)	85 (88.5)
Other	19 (27.1)	51 (15.7)	22 (31.4)	37 (12.5)	4 (16.0)	11 (11.5)
Living with partner						
Yes	63 (90.0)	292 (90.1)	55 (78.6)	263 (88.6)	22 (88.0)	86 (89.6)
No or missing data	7 (10.0)	32 (9.9)	15 (21.4)	34 (11.4)	3 (12.0)	10 (10.4)
Maternal smoking in early pregnancy, cigarettes/d						
0	53 (75.7)	258 (79.6)	47 (67.1)	245 (82.5)	20 (80.0)	69 (71.9)
1-9	8 (11.4)	27 (8.3)	4 (5.7)	22 (7.4)	3 (12.0)	16 (16.7)
≥10	5 (7.1)	18 (5.6)	11 (15.7)	15 (5.1)	2 (8.0)	6 (6.3)
Missing	4 (5.7)	21 (6.5)	8 (11.4)	15 (5.1)	0	5 (5.2)
Parity						
0	28 (40.0)	149 (46.0)	26 (37.1)	127 (42.8)	11 (44.0)	36 (37.5)
1	27 (38.6)	106 (32.7)	21 (30.0)	112 (37.7)	9 (36.0)	37 (38.5)
≥2	15 (21.4)	69 (21.3)	23 (32.9)	58 (19.5)	5 (20.0)	23 (24.0)
Maternal BMI at first visit						
Median (IQR) [range]	24.2 (21.6-27.9) [16.5-47.3]	23.3 (21.5-26.5) [16.6-47.4]	25.3 (23.2-29.1) [19.0-37.0]	23.3 (21.3-26.8) [17.1-38.1]	24.3 (21.5-31.0) [18.6-33.7]	22.4 (20.9-25.6) [16.9-39.7]
<18.5	2 (2.9)	5 (1.5)	0	5 (1.7)	0	4 (4.2)
18.5 to <25	29 (41.4)	163 (50.3)	27 (38.6)	160 (53.9)	10 (40.0)	52 (54.2)
25 to <30	16 (22.9)	67 (20.7)	21 (30.0)	63 (21.2)	4 (16.0)	18 (18.8)
≥30	10 (14.3)	26 (8.0)	10 (14.3)	20 (6.7)	6 (24.0)	3 (3.1)
Missing	13 (18.6)	63 (19.4)	12 (17.1)	49 (16.5)	5 (20.0)	19 (19.8)
GA at birth, wk^b^						
Median (IQR) [range]	39.7 (38.9-40.9) [30.1-42.6]	40.1 (39.0-40.9) [33.4-42.9]	39.9 (38.9-40.6) [30.1-42.7]	40.1 (39.0-41.0) [31.7-43.6]	40.0 (39.4-40.9) [31.0-42.0]	40.1 (39.1-40.8) [34.9-42.9]
Preterm	10 (14.3)	14 (4.3)	3 (4.3)	15 (5.1)	2 (8.0)	5 (5.2)
Full term	58 (82.9)	289 (89.2)	62 (88.6)	258 (86.9)	22 (88.0)	83 (86.5)
Postterm	2 (2.9)	21 (6.5)	5 (7.1)	24 (8.1)	1 (4.0)	8 (8.3)
Birth weight^c^						
Median (IQR) [range], g	3340 (3115-3806) [850-4410]	3590 (3270-3900) [1845-5180]	3373 (3060-3695) [1195-4515]	3563 (3265-3945) [2019-4940]	3355 (3107-3780) [1160-4530]	3690 (3275-4000) [2217-4940]
Low	8 (11.4)	7 (2.2)	3 (4.3)	7 (2.4)	3 (12.0)	2 (2.1)
Reference	51 (72.9)	249 (76.9)	58 (82.9)	227 (76.4)	18 (72.0)	69 (71.9)
High	10 (14.3)	66 (20.4)	9 (12.9)	62 (20.9)	4 (16.0)	24 (25.0)
Missing	1 (1.4)	2 (0.6)	0	1 (0.3)	0	1 (1.0)
Birth weight for GA^d^						
SGA	13 (18.6)	27 (8.3)	16 (22.9)	26 (8.8)	6 (24.0)	7 (7.3)
AGA	52 (74.3)	266 (82.1)	50 (71.4)	237 (79.8)	17 (68.0)	80 (83.3)
LGA	4 (5.7)	29 (9.0)	4 (5.7)	33 (11.1)	2 (8.0)	8 (8.3)
Missing	1 (1.4)	2 (0.6)	0	1 (0.3)	0	1 (1.0)
Mode of delivery						
Vaginal noninstrumental	55 (78.6)	247 (76.2)	61 (87.1)	232 (78.1)	22 (88.0)	75 (78.1)
Vaginal instrumental^e^	4 (5.7)	28 (8.6)	0	24 (8.1)	1 (4.0)	9 (9.4)
Cesarean	11 (15.7)	49 (15.1)	9 (12.9)	41 (13.8)	2 (8.0)	12 (12.5)
Maternal complications						
Pregestational diabetes	0	3 (0.9)	1 (1.4)	0	0	0
Gestational diabetes	0	4 (1.2)	4 (5.7)	1 (0.3)	0	2 (2.1)
Preeclampsia	6 (8.6)	18 (5.6)	9 (12.9)	25 (8.4)	5 (20.0)	5 (5.2)
Maternal diabetes	0	2 (0.6)	1 (1.4)	0	0	0
**At date of MASLD diagnosis or matching**						
Sex						
Female	33 (47.1)	152 (46.9)	25 (35.7)	103 (34.7)	7 (28.0)	30 (31.3)
Male	37 (52.9)	172 (53.1)	45 (64.3)	194 (65.3)	18 (72.0)	66 (68.8)
Age at diagnosis, median (IQR) [range], y	2.9 (0.8-8.1) [0.1-10.9]	2.9 (0.9-7.8) [0.0-11.0]	14.1 (12.4-16.7) [11.0-18.0]	14.0 (12.6-16.7) [11.1-18.0]	20.1 (18.4-21.9) [18.1-24.1]	20.0 (18.4-22.2) [18.0-24.0]
Year of diagnosis						
1992-1999	18 (25.7)	84 (25.9)	0	0	0	0
2000-2010	37 (52.9)	170 (52.5)	18 (25.7)	90 (30.3)	3 (12.0)	4 (4.2)
2011-2016	15 (21.4)	70 (21.6)	52 (74.3)	207 (69.7)	22 (88.0)	92 (95.8)
Parents’ highest educational level, y^f^						
≤9	4 (5.7)	5 (1.5)	7 (10.0)	6 (2.0)	0	2 (2.1)
10-12	28 (40.0)	133 (41.0)	34 (48.6)	128 (43.1)	11 (44.0)	42 (43.8)
≥13	38 (54.3)	186 (57.4)	29 (41.4)	163 (54.9)	14 (56.0)	52 (54.2)
MASLD histologic findings						
Simple steatosis	24 (34.3)	NA	23 (32.9)	NA	15 (60.0)	NA
MASH without fibrosis	12 (17.1)	NA	11 (15.7)	NA	4 (16.0)	NA
MASH with fibrosis	31 (44.3)	NA	34 (48.6)	NA	6 (24.0)	NA
Cirrhosis	3 (4.3)	NA	2 (2.9)	NA	0	NA
Comorbidities						
Cardiovascular disease	7 (10.0)	3 (0.9)	5 (7.1)	5 (1.7)	1 (4.0)	5 (5.2)
Diabetes	1 (1.4)	0	7 (10.0)	3 (1.0)	1 (4.0)	0
Hypertension	2 (2.9)	0	2 (2.9)	0	0	0
Dyslipidemia	0	0	0	0	0	0

Abbreviations: AGA, appropriate for gestational age; BMI, body mass index (calculated as weight in kilograms divided by height in meters squared); GA, gestational age; LGA, large for gestational age; MASH, metabolic dysfunction–associated steatohepatitis; MASLD, metabolic dysfunction–associated steatotic liver disease; NA, not assessed; SGA, small for gestational age.

^a^ Data are presented as number (percentage) of participants unless otherwise indicated. Children were aged 10 years or younger; adolescents, 11 to 17 years; and young adults, 18 to 25 years.

^b^ Preterm, less than 37 weeks; full term, 37 to 41 weeks; postterm, 42 or more weeks.

^c^ Low birth weight, 1500 g to less than 2500 g; reference, 2500 g to less than 4000 g; high, 4000 g or more.

^d^ SGA, less than 10th percentile; AGA, 10th to 90th percentile; LGA, greater than 90th percentile.

^e^ Vaginal instrumental delivery refers to either vacuum extraction or forceps delivery.

^f^ Categories correspond to compulsory school, high school, and college.

### Birth Anthropometrics and Odds of MASLD

**Figure 1.**
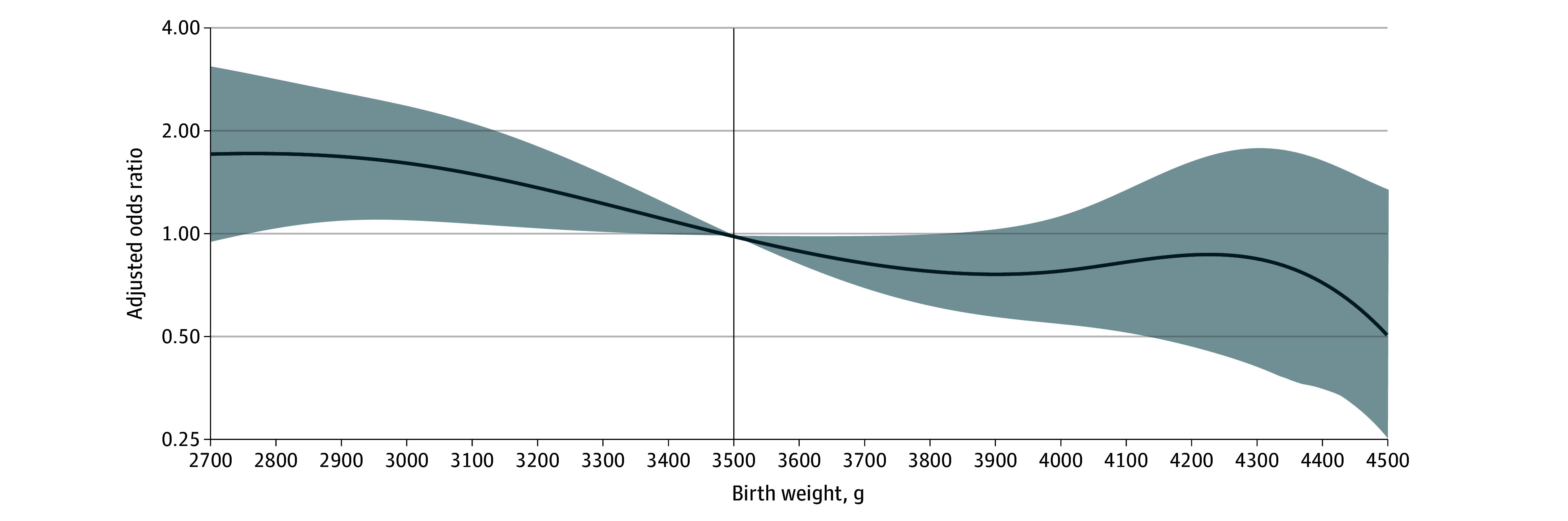
Association Between Birth Weight and Odds of Metabolic Dysfunction–Associated Steatotic Liver Disease (MASLD) Odds of MASLD according to birth weight are shown as a continuous variable using a restricted cubic spline function (reference birth weight, 3500 g). Shading indicates 95% CIs.

**Figure 2.**
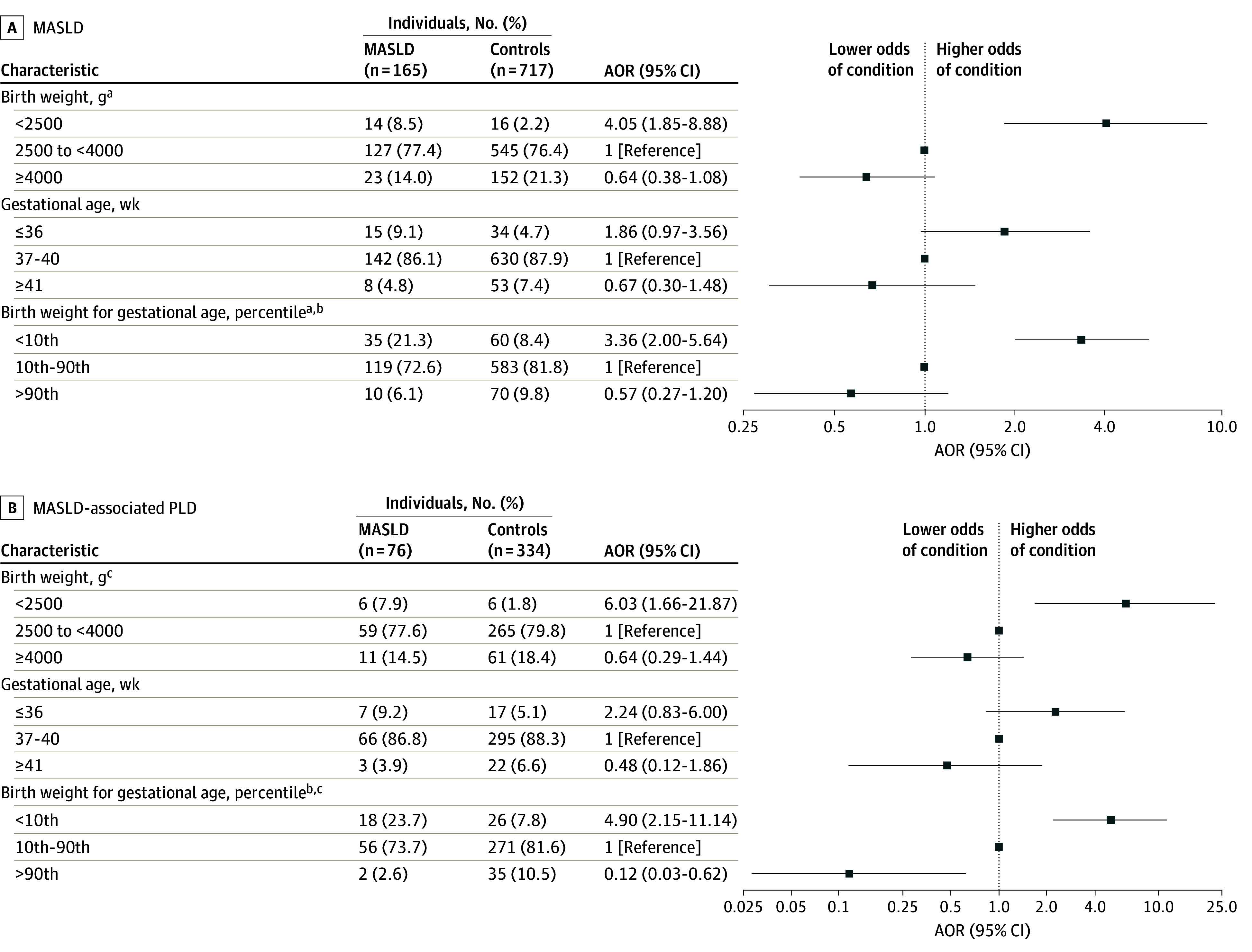
Odds of Metabolic Dysfunction–Associated Steatotic Liver Disease (MASLD) and Progressive Liver Disease (PLD) According to Birth Anthropometrics Logistic regression was conditioned on matching factors (age, sex, calendar year, and county of residence) and further adjusted for known or suspected maternal factors that may affect the risk for developing MASLD in offspring: maternal age, maternal early-pregnancy body mass index, maternal country of birth, parity, parents’ highest educational level, and smoking in early pregnancy. AOR indicates adjusted odds ratio. ^a^The total number of individuals with MASLD is 164 and of controls is 713. ^b^Birth weight below the 10th percentile was considered small for gestational age; 10th to 90th percentile, appropriate for gestational age; and greater than 90th percentile, large for gestational age. ^c^The total number of controls is 332.

Individuals with MASLD had a lower median birth weight compared with matched controls (3350 g [IQR, 3090-3773 g] vs 3590 g [IQR, 3265-3945 g]; *P* < .001). There was an inverse association between birth weight and odds of MASLD, with an AOR of 0.92 (95% CI, 0.89-0.95) for each additional 100 g of birth weight (Figure 1). Compared with those born with the reference birth weight, low birth weight was associated with higher odds of developing MASLD, with an AOR of 4.05 (95% CI, 1.85-8.88). Conversely, the proportion of individuals born with high birth weight was lower among individuals with MASLD compared with controls (23 [13.9%] vs 152 [21.2%]), with a corresponding AOR of 0.64 (95% CI, 0.38-1.08) indicating no association of high birth weight with MASLD. While GA was not associated with MASLD, being born SGA but not LGA was associated with higher odds of future MASLD (SGA: AOR, 3.36; 95% CI, 2.00-5.64; LGA: AOR, 0.57; 95% CI, 0.27-1.20) compared with those born with size appropriate for GA (Figure 2A).

### Birth Anthropometrics and Odds of Progressive Liver Disease

In total, 76 individuals with MASLD (46.1%) met the criteria for progressive liver disease with histologic evidence of either liver fibrosis or cirrhosis. The odds of progressive liver disease were increased among individuals with low birth weight (AOR, 6.03; 95% CI, 1.66-21.87) and individuals born SGA (AOR, 4.90; 95% CI, 2.15-11.14). Gestational age was not associated with the development of progressive liver disease (Figure 2B). In contrast, being born LGA was associated with lower odds of progressive liver disease, with an AOR of 0.12 (95% CI, 0.03-0.62), compared with being born with appropriate size for GA.

### Sensitivity and Subgroup Analyses

When the logistic regression analyses were additionally adjusted for the metabolic morbidities of pregnancy, preeclampsia and gestational diabetes, the estimates remained largely unchanged (eTable 4 in Supplement 1). The findings were furthermore robust after restricting the analysis to those older than 2 years at time of MASLD diagnosis to address potential residual confounding related to suspected or undiagnosed inborn errors of metabolism (eTable 5 in Supplement 1) as well as after restricting the analysis to individuals diagnosed since 2004 (eTable 6 in Supplement 1). Subgroup analyses revealed that the association of birth weight with odds of pediatric-onset MASLD was independent of sex (eTable 7 in Supplement 1).

### Sibling-Controlled Analyses

To disentangle genetic factors associated with MASLD disease development and to account for shared early childhood exposures, individuals with biopsy-proven MASLD were compared with their full siblings. A total of 108 individuals with MASLD had at least 1 living full sibling. In the comparison of full siblings with general population controls, siblings also had a lower median birth weight (3423 g [IQR, 3140-3810 g] vs 3590 g [IQR, 3265-3945 g]), but there was no difference in birth weight between individuals who were diagnosed with pediatric-onset MASLD and their full siblings (3405 g [IQR, 3105-3773 g] vs 3423 g [IQR, 3140-3810 g]) (eTable 8 in Supplement 1). After adjustment for multiple confounders, AORs were above 1.00, but there was no association of low birth weight (AOR, 1.73; 95% CI, 0.46-6.55) and SGA (AOR, 2.56; 95% CI, 0.90-7.30) (eTable 9 in Supplement 1) with the development of MASLD.

## Discussion

We conducted a nationwide study of children, adolescents, and young adults with biopsy-proven MASLD and matched general population controls and found an inverse association between birth weight and odds of MASLD and progressive liver disease (defined as histologic evidence of liver fibrosis or cirrhosis). Individuals born with low birth weight and those born SGA had 3- to 4-fold increased odds of developing MASLD and 6-fold increased odds of developing progressive liver disease. The results were robust against adjustment for relevant maternal and perinatal factors to minimize any confounding and provide evidence for the developmental origins of MASLD and its complications on a population basis. In addition, we performed several sensitivity analyses that demonstrated the robustness of our findings and also minimized the risk of misclassification due to inborn errors of metabolism or changed definitions of MASLD.

In contrast, several previous studies did not find any association between birth anthropometrics and MASLD.^35,36,37,38,39,40,41^ However, when evaluating studies on MASLD, important limitations and considerations must be considered to properly compare results between studies. A major limitation of some previous studies is the lack of information on and, thus, adjustment for maternal factors, such as prepregnancy or early-pregnancy BMI, socioeconomic status, or development of gestational diabetes, all of which have been shown to be associated with obesity, cardiometabolic disease, and MASLD in offspring.^42,43,44^ However, maternal factors are associated with birth weight, with a higher risk of offspring being born LGA among mothers with obesity.^45^ Therefore, studies that do not adjust for maternal prepregnancy BMI may be more likely to underestimate or miss associations with low birth weight or SGA while overestimating associations with high birth weight or LGA or even reporting false-positive associations. In our study, the early-pregnancy BMI was higher in mothers whose offspring were later diagnosed with MASLD compared with mothers of controls (25.0 vs 23.1; *P* < .001).

Another factor that needs to be considered when comparing studies on the developmental origins of MASLD is the age at the time of MASLD diagnosis. It appears that pediatric-onset MASLD is associated with a stronger inflammatory burden and a more aggressive course of disease than adult-onset MASLD, which mostly arises from decades of obesity.^46^ Hence, studies in older populations may simply mirror associations of perinatal characteristics with the lifetime risk of obesity, while studies in younger populations may be able to detect determinants for adverse metabolic programming associated with hepatic fat accumulation, inflammation, and periportal fibrogenesis.^47^ The same bias applies to studies using noninvasive scoring systems to detect MASLD, as most equations are usually not specific for MASLD but are primarily determined by the individual’s BMI and waist circumference; thus, the results apply more to associations with future risk of obesity than to associations with risk of MASLD. For example, in a recent large, prospective, French cohort study of young adults^48^ assessed using the Fatty Liver Index,^49^ the authors found a U-shaped association between birth weight and future odds of MASLD, which would be more in line with previous evidence on obesity but contradicts our findings showing no associations between LGA and MASLD and lower odds of developing progressive liver disease. Our results are consistent with some previous studies that likewise found an inverse association between birth weight and MASLD instead of a U shape.^50,51^ The large Cardiovascular Risk in Young Finns Study prospectively observed 2042 individuals who underwent liver ultrasonography and found lower odds of MASLD associated with increasing birth weight (OR, 0.77; 95% CI, 0.68-0.88).^50^ In addition, a cross-sectional study on 288 individuals with biopsy-proven MASLD from Italy also found an inverse association of birth weight with the histologic degrees of steatosis, inflammation, and fibrosis.^51^

### Clinical Implications

The steady increase in the global prevalence of MASLD in young people is particularly concerning as early-onset MASLD persisting until adulthood is associated with a high risk of developing cirrhosis or end-stage liver disease requiring liver transplant or ultimately dying from liver-related causes.^8,30^ Furthermore, these individuals are at markedly increased risk of developing cardiovascular disease, including major adverse cardiovascular events and heart failure.^52^ In our study, a significant proportion of young individuals with MASLD already had clinical evidence of cardiovascular disease. Against the background of the high burden of disease associated with pediatric-onset MASLD, effective strategies are essential to identify individuals at high risk for progressive liver disease to provide them effective preventive measures and surveillance. Because MASLD develops and progresses silently, affected people are asymptomatic, which is why among young individuals, it is most often diagnosed incidentally through screening.^53^ Current guidelines recommend screening for MASLD in all young people diagnosed with obesity,^54^ as previous therapeutic interventions to treat childhood obesity have consistently been more effective when implemented in early childhood compared with puberty or after.^55^ The high prevalence and long asymptomatic natural history of MASLD, with relevant long-term health risks, make the disease highly amenable to screening. However, guideline recommendations are largely based on low-quality evidence, and many questions remain unanswered. Our findings provide population-based evidence highlighting that small, vulnerable newborns^56,57^ (ie, individuals with low birth weight or SGA) should receive early, structured screening for MASLD to diagnose MASLD at an early stage and establish lifestyle interventions to prevent progression to liver fibrosis and cirrhosis. Every fourth neonate worldwide is born either too soon or too small,^58^ and the WHO has recommended effective measures for prevention^59,60^ and called for action to reduce the number of and health care burden for small, vulnerable newborns.^57^

### Strengths and Limitations

This study has several strengths. First, the well-characterized nationwide cohort at the population level ensured high external validity of the estimates. Second, data on liver histologic findings allowed us to specifically investigate the association of birth weight, GA, and birth weight for GA with the development of progressive liver disease. Third, due to the tax-funded universal Swedish health care system, which results in a high uptake of antenatal care by almost all pregnant individuals in Sweden, the MBR has been shown to have high data quality and accuracy.^29^ Fourth, we rigorously excluded any other known causes of hepatic steatosis, including inborn errors of metabolism, and we adjusted all analyses for well-established confounders, including demographics, socioeconomic status, clinical parameters, and pregnancy-related morbidities, thereby minimizing any confounding. In addition, the identification of all full siblings of individuals with MASLD allowed for sibling-controlled analyses that accounted for genetic and early environmental factors.

Nevertheless, our study has limitations. It must be interpreted in the context of the study design. First, despite careful matching and adjustment for various demographic and clinical confounders, residual confounding cannot be completely excluded, especially since we did not have data on weight trajectories through infancy and childhood. As such, we lacked information on whether mothers chose to breastfeed or not, which has been suggested to reduce the risk of MASLD and progressive liver disease in offspring.^61,62^ Second, despite the population-based design of our study, some analyses may have been underpowered. Also, since our study was based on biopsy-proven MASLD, there was an inherent risk of selection bias toward individuals with clinical suspicion of advanced liver disease, as most young individuals with MASLD do not undergo liver biopsy. Third, we were not able to adjust for ethnic background, as Swedish registries do not collect information on ethnicity by law, but we addressed this issue by using maternal country of birth as a proxy. Fourth, as familial and twin studies have suggested a high prevalence of MASLD in full siblings,^63,64^ it can be assumed that a relevant proportion of siblings in our study actually had undiagnosed MASLD, which is in line with their lower birth weight compared with general population controls. However, the MASLD status of siblings was not systematically ascertained, so the sibling-controlled analyses must be interpreted with caution.

## Conclusions

This nationwide case-control study of children, adolescents, and young adults with biopsy-proven MASLD found higher odds of MASLD and progressive liver disease in small, vulnerable newborns (ie, born with low birth weight or SGA). The study findings suggest that MASLD and MASLD-associated progressive liver disease have developmental origins, and it highlights the need for intensified preventive measures to reduce the number of small, vulnerable newborns and to implement structured screening measures to diagnose MASLD-associated progressive liver disease early in high-risk individuals.

## Notes

Supplement 1. **eFigure.** Study Flowchart **eTable 1.** Study Exclusion Criteria **eTable 2.** Definition of Baseline Covariates in Mothers **eTable 3.** Characteristics of MASLD Individuals and Controls at Birth and at Index Date **eTable 4.** Sensitivity Analyses on Odds of MASLD and Progressive Liver Disease **eTable 5.** Odds of MASLD in Restricted Cohort Aged 2 Years or Older at Time of MASLD Diagnosis **eTable 6.** Odds of MASLD in Restricted Cohort for Individuals Diagnosed With MASLD From 2004 Onward **eTable 7.** Odds of MASLD Among Females and Males **eTable 8.** Characteristics of MASLD Individuals and Their Siblings at Birth and at Index Date **eTable 9.** Birth Weight, Gestational Age, and Future Odds of MASLD in Sibling-Controlled Analysis
eReferences

Supplement 2.
Data Sharing Statement
