# Supplementary material for: Birth Weight, Gestational Age, and Risk of Pediatric-Onset MASLD
Source: JAMA Netw Open. 2024 Sep 10;7(9):e2432420. doi: 10.1001/jamanetworkopen.2024.32420 (PMC11388034; doi:10.1001/jamanetworkopen.2024.32420)
Supplement: Supplement 1. — eFigure. Study Flowchart eTable 1. Study Exclusion Criteria eTable 2. Definition of Baseline Covariates in Mothers eTable 3. Characteristics of MASLD Individuals and Controls at Birth and at Index Date eTable 4. Sensitivity Analyses on Odds of MASLD and Progressive Liver Disease eTable 5. Odds of MASLD in Restricted Cohort Aged 2 Years or Older at Time of MASLD Diagnosis eTable 6. Odds of MASLD in Restricted Cohort for Individuals Diagnosed With MASLD From 2004 Onward eTable 7. Odds of MASLD Among Females and Males eTable 8. Characteristics of MASLD Individuals and Their Siblings at Birth and at Index Date eTable 9. Birth Weight, Gestational Age, and Future Odds of MASLD in Sibling-Controlled Analysis eReferences [file jamanetwopen-e2432420-s001.pdf]

Supplementary Online Content

Ebrahimi F, Yao J, Hagström H, et al. Birth weight, gestational age, and risk of pediatric-onset MASLD. *JAMA Netw Open*. 2024;7(9):e2432420.
doi:10.1001/jamanetworkopen.2024.32420

eFigure. Study Flow Chart

eTable 1. Study Exclusion Criteria

eTable 2. Definition of Baseline Covariates in Mothers

eTable 3. Characteristics of MASLD Individuals and Controls at Birth and at Index Date

eTable 4. Sensitivity Analyses on Odds of MASLD and Progressive Liver Disease

eTable 5. Odds of MASLD in Restricted Cohort Aged 2 Years or Older at Time of MASLD Diagnosis

eTable 6. Odds of MASLD in Restricted Cohort for Individuals Diagnosed With MASLD From 2004 Onward

eTable 7. Odds of MASLD Among Females and Males

eTable 8. Characteristics of MASLD Individuals and Their Siblings at Birth and at Index Date

eTable 9. Birth Weight, Gestational Age, and Future Odds of MASLD in Sibling-Controlled Analysis

eReferences

This supplementary material has been provided by the authors to give readers additional information about their work.

© 2024 Ebrahimi F et al. *JAMA Network Open*.

eFigure. Study Flow Chart

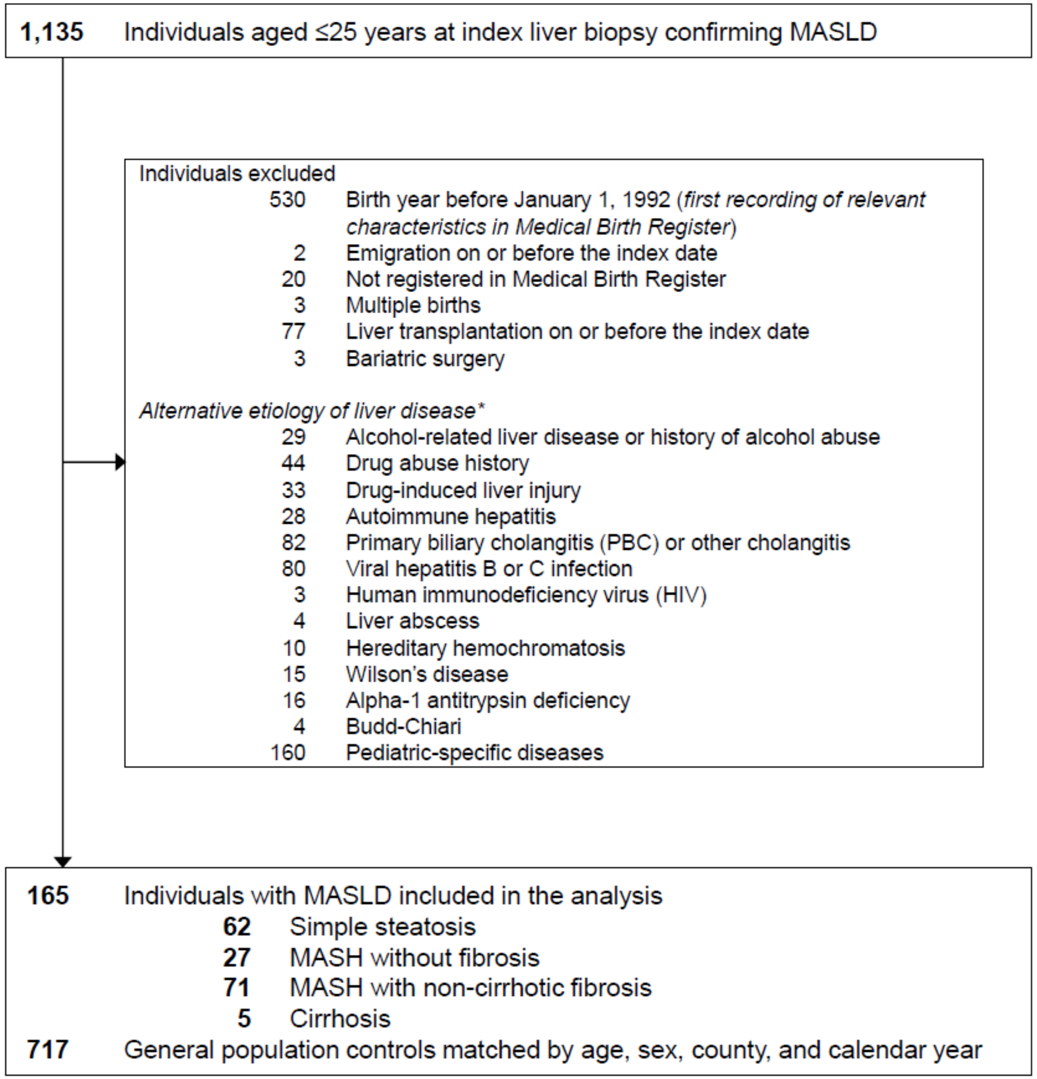

*Some individuals had multiple concomitant liver diseases, thus the total number of alternative etiologies of liver diseases exceeds the sum of excluded individuals.
Abbreviations: MASLD, metabolic dysfunction-associated steatotic liver disease; MASH, metabolic dysfunction-associated steatohepatitis.

© 2024 Ebrahimi F et al. *JAMA Network Open*.

eTable 1. Study Exclusion Criteria

Conditions	ICD-7 / 8	ICD-9	ICD-10
Alcohol abuse / misuse, or Alcohol-related liver disease	280,00; 281,00; 307,00; 307,10; 307,99; 322; 581,10; 583,10; 261,00; 262,00; 291; 291,1; 303; 571,00; 571,01; 979; 980,00; 980,01; 980,98; 980,99	291; 294A; 303; 305A; 357F; 425F; 535D; 571A-D; 760W; 790D; 977D; 980A; 980X; V97B	E24.4; F10; G31.2; G62.1; G72.1; I42.6; K29.2; K70; K86.0; Q35.4; R78.0; T51.0; T51.8; T51.9; X65; Y15; Y57.3; Y90; Y91; Z50.2; Z71.4; Z71.2
Other drug abuse	5710, E860, N980	571A-D	F11-F19
Drug-induced liver disease	–	573D	K71
Viral hepatitis B or C infection	070	70; 070A-G; 070X	B15-19, B16.0, B16.1, B16.2, B16.9, B17.0-B17.9, B18.0-B18.9, B19.0-B19.9; B00.8; B25.1
Budd-Chiari	–	453A	I82
Liver abscess	572; 572,00	5720	K75.0, A06.4
HIV		279K	B20-B24
Hemochromatosis	273,2	275A	E83.1
Wilson’s disease	273,3	275B	E83.0
Autoimmune hepatitis	–	571.42	K75.4
Primary biliary cholangitis	–	571G	K74.3, K74.4
Primary sclerosing cholangitis /Other cholangitis	574,06	576B	K83; K83.0A
Alpha-1 antitrypsin deficiency	–	273E; 277G; 274E; 573W	E88.01
Liver transplantation	Swedish procedure codes: 5200, 5202, JJC00, JJC10, JJC20, JJC30, JJC		

Pediatric-specific Exclusions	ICD-9	ICD-10
Glycogen storage disease / disorders of carbohydrate metabolism	271 (except 271A, 271D)	E73.0; E74
Other congenital disorders of glycosylation	271A	E74
Lipid or lipoprotein storage or glycoprotein metabolism disorders	272; 330B	E75 to E78
Mitochondrial disease / disorders of respiratory chain or fatty acid metabolism or plasma protein metabolism defects	277F; 330A; 330W	G31.8; E71.3; E88
Amino acid metabolism / storage disorders, urea cycle defects, tyrosinemia, citrin deficiencies	270	E70; E71; E72; Q87.8D
Peroxisomal diseases	277	E88.8
Porphyria	277B	E80
Cystic fibrosis	277A	E84
Cushing syndrome	255A	E24
Polycystic ovarian syndrome	256E	E28.2
Polyglandular syndromes	258	E31
Lipodystrophy	272G	E88.1
Malnutrition	260-263; 307B	E40-46; E64.0; F50.0; F50.1
Prader-Willi Syndrome / Other congenital liver malformations	759H; 759W; 759X	Q87.1; Q447

© 2024 Ebrahimi F et al. *JAMA Network Open*.

Turner Syndrome	758G	Q96
Congenital leukocyte disorders (including Schwachman-Diamond syndrome)	288C	D70.9D

**Excluded Medications[1]**	**ATC Code (Prescribed Drug Register)**
Systemic steroids	01AC02, C05AA09, D07AB19, D10AA03, H02AB02, R01AD03, S01BA01, S01CB01, S02BA06, S03BA01, H02AB15, A01AC03, A07EA02, C05AA01, D07AA02, D07XA01, H02AB09, S01BA02, S01CB03, S02BA01, D07AC16, D07AB11, D07AB02, D07AC16, A07AE03, H02AB07
Tamoxifen	L02BA01
Methotrexate	L01BA01, L04AX03
Interferon	L03AB
Direct-acting antiviral therapy	JO5, JO5A, JO5AA-AH, JO5AP, JO5AR
(Non-)Nucleos(t)ide Reverse Transcriptase Inhibitors	J05AF, J05AG
Valproic acid	N03AG01
Amiodarone	C01BD01
Systemic steroids	01AC02, C05AA09, D07AB19, D10AA03, H02AB02, R01AD03, S01BA01, S01CB01, S02BA06, S03BA01, H02AB15, A01AC03, A07EA02, C05AA01, D07AA02, D07XA01, H02AB09, S01BA02, S01CB03, S02BA01, D07AC16, D07AB11, D07AB02, D07AC16, A07AE03, H02AB07
Tamoxifen	L02BA01
Methotrexate	L01BA01, L04AX03
Interferon	L03AB

All ICD codes are based on the Swedish version of the ICD system.

[1]We excluded any person with prior use of a steatogenic medication or a medication used to treat an alternative etiology of liver disease. Medication use was defined by a filled prescription for at least 30 cumulative defined daily doses (cDDD), in the Prescribed Drug Register, at any time prior to the index date (or corresponding matching date), with the exception of systemic steroids (for which a person was excluded if they used systemic steroids within 0 to 3 months prior to the index date).

Abbreviations: ATC, anatomic therapeutic chemical classification; HIV, human immunodeficiency virus; ICD, International Classification of Disease.

© 2024 Ebrahimi F et al. *JAMA Network Open*.

eTable 2. Definition of Baseline Covariates in Mothers

**Baseline covariates** (up until and including date of biopsy/matching date)					
	**ICD-8 (1969-1986)**	**ICD-9 (1987-1996)**	**ICD-10 (1997-)**	**Medication (ATC)**	**Notes**
**Comorbidities**					
Pregestational diabetes mellitus	-	250; 648A	O240-O243	-	Diagnoses according to Medical Birth Register.
Gestational diabetes	-	648W	O244	-	Medical Birth Register.
Obesity	-	278; 649,1; 649B	E65-E67	-	ICD code **OR** Maternal BMI ≥30kg/m² from Medical Birth Register.
Primary hypertension	-	401-405; 642C	I10-I15; O10-O11;	C09A-C09D; C09X	Medical Birth Register and Prescribed Drug Register.
Gestational hypertension	-	642D; 642X	O13	-	Medical Birth Register
Smoking status	-	-	-	-	Medical Birth Register: 0=missing; 1=non-smoker; 2=1–9 cig/day; 3 ≥10 cig/day

Abbreviations: ICD, International Classification of Disease; ATC, anatomic therapeutic chemical classification.

© 2024 Ebrahimi F et al. *JAMA Network Open*.

eTable 3. Characteristics of MASLD Individuals and Controls at Birth and at Index date

Characteristic	MASLD (n=165)	Controls (n=717)	*p* value
**Characteristics at births**			
Maternal age (years)			
Median (IQR)	28.7 (25.0-33.5)	29.7 (26.0-33.0)	0.36
Range, min-max	17.8-42.8	17.5-44.8	
Categories, n (%)			
≤24	41 (24.8%)	128 (17.9%)	
25-29	47 (28.5%)	253 (35.3%)	0.11
30-34	45 (27.3%)	214 (29.8%)	
≥35	32 (19.4%)	122 (17.0%)	
Birth year, n (%)			
1992-1999	102 (61.8%)	435 (60.7%)	
2000-2010	61 (37.0%)	273 (38.1%)	0.96
2011-2016	2 (1.2%)	9 (1.3%)	
Maternal country of birth, n (%)			
Nordic country	120 (72.7%)	618 (86.2%)	<0.001
Other	45 (27.3%)	99 (13.8%)	
Living with partner			
Yes	140 (84.8%)	641 (89.4%)	0.10
No/missing	25 (15.2%)	76 (10.6%)	
Maternal smoking in early pregnancy			
Non-smoking	120 (72.7%)	572 (79.8%)	
1-9 cig/day	15 (9.1%)	65 (9.1%)	0.06
≥10 cig/day	18 (10.9%)	39 (5.4%)	
Missing	12 (7.3%)	41 (5.7%)	
Parity			
0	65 (39.4%)	312 (43.5%)	
1	57 (34.5%)	255 (35.6%)	0.33
≥2	43 (26.1%)	150 (20.9%)	
Maternal BMI at first visit (kg/m2)			
Median (IQR)	25.0 (22.0-29.0)	23.1 (21.3-26.5)	<0.001
Range, min-max	16.5; 47.3	16.6; 47.4	
Categories, n (%)			
<18.5	2 (1.2%)	14 (2.0%)	
18.5 - <25	66 (40.0%)	375 (52.3%)	
25 - <30	41 (24.8%)	148 (20.6%)	<0.001
≥30	26 (15.8%)	49 (6.8%)	
Missing	30 (18.2%)	131 (18.3%)	
Birthweight (gram), n (%)			
Median (IQR)	3350 (3090-3773)	3590 (3265-3945)	<0.001
Range, min-max	850-4530	1845-5180	
Categories, n (%)			
Low birthweight (1500-<2500g)	14 (8.5%)	16 (2.2%)	
Normal birthweight (2500-<4000g)	127 (77.0%)	545 (76.0%)	
High birthweight (≥4000g)	23 (13.9%)	152 (21.2%)	
Missing	1 (0.6%)	4 (0.6%)	
Gestational age at birth (weeks), n (%)			
Median (IQR)	39.9 (38.9-40.7)	40.1 (39.0-41.0)	0.08
Range, min-max	30.1-42.7	31.7-43.6	
Categories, n (%)			
Preterm (<37 weeks)	15 (9.1%)	34 (4.7%)	0.03
Full term (37-41 weeks)	142 (86.1%)	630 (87.9%)	0.53
Post term (≥42 weeks)	8 (4.8%)	53 (7.4%)	0.25
Birthweight for gestational age			
SGA (<10th percentile)	35 (21.2%)	60 (8.4%)	
AGA (10-90th percentile)	119 (72.1%)	583 (81.3%)	<0.001
LGA (>90th percentile)	10 (6.1%)	70 (9.8%)	
Missing	1 (0.6%)	4 (0.6%)	
Mode of delivery, n (%)*			
Vaginal non-instrumental delivery	138 (83.6%)	554 (77.3%)	
Vaginal instrumental delivery	5 (3.0%)	61 (8.5%)	0.05
Caesarean section	22 (13.3%)	102 (14.2%)	

© 2024 Ebrahimi F et al. *JAMA Network Open*.

Maternal complications			
Pre-gestational diabetes, n (%)	1 (0.6%)	3 (0.4%)	0.75
Gestational diabetes, n (%)	4 (2.4%)	7 (1.0%)	0.13
Pre-eclampsia, n (%)	20 (12.1%)	48 (6.7%)	0.02
**Characteristics in study subjects at date of MASLD diagnosis/matching**			
Sex, n (%)			
Female	65 (39.4%)	285 (39.7%)	0.93
Male	100 (60.6%)	432 (60.3%)	
Age at diagnosis/index date (years)			
Median (IQR)	12.0 (4.4-16.9)	11.7 (3.4-16.3)	0.51
Range, min-max	0.1-24.1	0.0-24.0	
Categories, n (%)			
Children (≤10y)	70 (42.4%)	324 (45.2%)	
Adolescents (11-17y)	70 (42.4%)	297 (41.4%)	0.75
Young adults (18-25y)	25 (15.2%)	96 (13.4%)	
Year at diagnosis/index date, n (%)			
1992-1999	18 (10.9%)	84 (11.7%)	
2000-2010	58 (35.2%)	264 (36.8%)	0.85
2011-2016	89 (53.9%)	369 (51.5%)	
Highest level of education in parents[#]			
≤9 years	11 (6.7%)	13 (1.8%)	
10 - 12 years	73 (44.2%)	303 (42.3%)	0.002
≥13 years	81 (49.1%)	401 (55.9%)	
MASLD histology, n (%)			
Simple steatosis	62 (37.6%)	–	–
MASH without fibrosis	27 (16.4%)	–	–
MASLD with non-cirrhotic fibrosis	71 (43.0%)	–	–
Cirrhosis	5 (3.0%)	–	–
Comorbidities, n (%)			
Cardiovascular Disease	13 (7.9%)	13 (1.8%)	<0.001
Diabetes mellitus	9 (5.5%)	3 (0.4%)	<0.001
Hypertension	4 (2.4%)	0	<0.001
Dyslipidemia	0	0	-

*Vaginal instrumental delivery refers to either vacuum extraction or forceps delivery.
[#]Education categories based on compulsory school, high school, and college.
Abbreviations: AGA, appropriate for gestational age; BMI, body mass index; LGA, large for gestational age; MASLD, metabolic dysfunction-associated steatotic liver disease; MASH, metabolic dysfunction-associated steatohepatitis; SGA, small for gestational age.

© 2024 Ebrahimi F et al. *JAMA Network Open*.

eTable 4. Sensitivity Analyses on Odds of MASLD and Progressive Liver Disease

Risk of MASLD	MASLD	Controls	aOR (95% CI)*
**Birthweight**			
N	164	713	
Low birthweight (<2500g)	14 (8.5%)	16 (2.2%)	3.41 (1.51-7.72)
Normal birthweight (2500-<4000g) (reference)	127 (77.4%)	545 (76.4%)	1.00
High birthweight (≥4000g)	23 (14.0%)	152 (21.3%)	0.63 (0.37-1.07)
**Gestational age**			
N	165	717	
Preterm (≤36 weeks)	15 (9.1%)	34 (4.7%)	1.58 (0.81-3.10)
Full term (37-41 weeks)	142 (86.1%)	630 (87.9%)	1.00
Post-term (≥42 weeks)	8 (4.8%)	53 (7.4%)	0.66 (0.30-1.48)
**Birthweight for gestational age**			
N	164	713	
SGA (<10th percentile)	35 (21.3%)	60 (8.4%)	3.12 (1.84-5.29)
AGA (10-90th percentile) (reference)	119 (72.6%)	583 (81.8%)	1.00
LGA (>90th percentile)	10 (6.1%)	70 (9.8%)	0.55 (0.26-1.18)
**Risk of Progressive Liver Disease**			
**Birthweight**			
N	76	332	
Low birthweight (<2500g)	6 (7.9%)	6 (1.8%)	4.24 (1.03-17.53)
Normal birthweight (2500-<4000g)	59 (77.6%)	265 (79.8%)	1.00
High birthweight (≥4000)	11 (14.5%)	61 (18.4%)	0.62 (0.27-1.40)
**Gestational age**			
N	76	334	
Preterm (≤36 weeks)	7 (9.2%)	17 (5.1%)	1.52 (0.50-4.60)
Full term (37-41 weeks)	66 (86.8%)	295 (88.3%)	1.00
Post-term (≥42 weeks)	3 (3.9%)	22 (6.6%)	0.45 (0.12-1.73)
**Birthweight for gestational age**			
N	76	332	
SGA (<10th percentile)	18 (23.7%)	26 (7.8%)	4.33 (1.85-10.10)
AGA (10-90th percentile) (reference)	56 (73.7%)	271 (81.6%)	1.00
LGA (>90th percentile)	2 (2.6%)	35 (10.5%)	0.12 (0.02-0.60)

*Conditional logistic regression matched for age at index date, sex, calendar year, and county of residence and further adjusted for maternal age, maternal early-pregnancy body mass index, maternal country of birth, parity, highest level of education in parents, and smoking in early pregnancy as well as pre-eclampsia and gestational diabetes.

Abbreviations: AGA, appropriate for gestational age; CI, Confidence interval; LGA, large for gestational age; MASLD, metabolic dysfunction-associated steatotic liver disease; aOR, adjusted odds ratio; SGA, small for gestational age.

© 2024 Ebrahimi F et al. *JAMA Network Open*.

eTable 5. Odds of MASLD in Restricted Cohort ≥2 years of Age at Time of MASLD Diagnosis

	MASLD	Controls	aOR (95% CI)*	aOR (95% CI)**
**Birthweight**				
N	136	588		
Low birthweight (<2500g)	9 (6.6%)	13 (2.2%)	2.67 (1.13-6.29)	2.82 (1.11-7.16)
Normal birthweight (2500-<4000g)	108 (79.4%)	455 (77.4%)	1.00	1.00
High birthweight (≥4000)	19 (14.0%)	120 (20.4%)	0.64 (0.38-1.10)	0.60 (0.33-1.07)
Birthweight as continuous variable by 100g increase	136	588	0.93 (0.90-0.97)	0.93 (0.89-0.96)
**Gestational age**				
N	136	592		
Preterm (≤36 weeks)	9 (6.6%)	27 (4.6%)	1.40 (0.66-2.98)	1.34 (0.60-2.99)
Term (37-41 weeks)	120 (88.2%)	519 (87.7%)	1.00	1.00
Post-term (≥42 weeks)	7 (5.1%)	46 (7.8%)	0.67 (0.29-1.53)	0.72 (0.30-1.72)
**Birthweight for gestational age**				
N	136	588		
SGA (<10th percentile)	26 (19.1%)	46 (7.8%)	2.66 (1.55-4.57)	3.14 (1.71-5.78)
AGA (10-90th percentile) (reference)	101 (74.3%)	484 (82.3%)	1.00	1.00
LGA (>90th percentile)	9 (6.6%)	58 (9.9%)	0.69 (0.33-1.45)	0.57 (0.25-1.29)

* Model I: Conditional logistic regression matched for age at index date, sex, calendar year, and county of residence.
**Model II: Model I and further adjusted for maternal age, maternal early-pregnancy body mass index, maternal country of birth, parity, highest level of education in parents, and smoking in early pregnancy.
Abbreviations: AGA, appropriate for gestational age; CI, Confidence interval; LGA, large for gestational age; MASLD, metabolic dysfunction-associated steatotic liver disease; aOR, adjusted odds ratio; SGA, small for gestational age.

© 2024 Ebrahimi F et al. *JAMA Network Open*.

eTable 6. Odds of MASLD in Restricted Cohort for Individuals diagnosed with MASLD from 2004 onwards

	MASLD	Controls	aOR (95% CI)*	aOR (95% CI)**
**Birthweight**				
N	136	578		
Very low or low birthweight (<2500g)	9 (6.6%)	12 (2.1%)	2.93 (1.23-7.02)	3.39 (1.32-8.69)
Normal birthweight (2500-<4000g)	106 (77.9%)	448 (77.5%)	1.00	1.00
High or very high birthweight (≥4000)	21 (15.4%)	118 (20.4%)	0.73 (0.44-1.22)	0.71 (0.40-1.25)
Birthweight as continuous variable by 100g increase	136	578	0.93 (0.90-0.96)	0.92 (0.89-0.96)
**Gestational age**				
N	136	582		
Preterm (≤36 weeks)	9 (6.6%)	25 (4.3%)	1.50 (0.70-3.22)	1.47 (0.65-3.30)
Term (37-41 weeks)	120 (88.2%)	509 (87.5%)	1.00	1.00
Post-term (≥42 weeks)	7 (5.1%)	48 (8.2%)	0.62 (0.27-1.40)	0.62 (0.26-1.46)
**Birthweight for gestational age**				
N	136	578		
SGA (<10th percentile)	29 (21.3%)	48 (8.3%)	2.94 (1.74-4.97)	3.37 (1.88-6.03)
AGA (10-90th percentile) (reference)	98 (72.1%)	473 (81.8%)	1.00	1.00
LGA (>90th percentile)	9 (6.6%)	57 (9.9%)	0.72 (0.34-1.50)	0.62 (0.28-1.40)

* Model I: Conditional logistic regression matched for age at index date, sex, calendar year, and county of residence.
**Model II: Model I and further adjusted for maternal age, maternal early-pregnancy body mass index, maternal country of birth, parity, highest level of education in parents, and smoking in early pregnancy.
Abbreviations: AGA, appropriate for gestational age; CI, Confidence interval; LGA, large for gestational age; MASLD, metabolic dysfunction-associated steatotic liver disease; aOR, adjusted odds ratio; SGA, small for gestational age.

© 2024 Ebrahimi F et al. *JAMA Network Open*.

eTable 7. Odds of MASLD among Females and Males

	MASLD	Controls	aOR (95% CI)*	aOR (95% CI)**
**Females**				
**Birthweight**				
N	65	284		
Low birthweight (<2500g)	6 (9.2%)	8 (2.8%)	3.31 (1.09-10.01)	3.93 (1.21-12.76)
Normal birthweight (2500-<4000g)	52 (80.0%)	230 (81.0%)	1.00	1.00
High birthweight (≥4000)	7 (10.8%)	46 (16.2%)	0.64 (0.27-1.53)	0.64 (0.25-1.66)
**Gestational age**				
N	65	285		
Preterm (≤36 weeks)	7 (10.8%)	19 (6.7%)	1.67 (0.68-4.08)	1.54 (0.61-3.90)
Term (37-41 weeks)	55 (84.6%)	246 (86.3%)	1.00	1.00
Post-term (≥42 weeks)	3 (4.6%)	20 (7.0%)	0.65 (0.19-2.27)	0.72 (0.19-2.65)
**Birthweight for gestational age**				
N	65	284		
SGA (<10th percentile)	16 (24.6%)	26 (9.2%)	3.14 (1.53-6.43)	4.82 (2.15-10.82)
AGA (10-90th percentile) (reference)	44 (67.7%)	228 (80.3%)	1.00	1.00
LGA (>90th percentile)	5 (7.7%)	30 (10.6%)	0.80 (0.29-2.24)	0.77 (0.25-2.37)
**Males**				
**Birthweight**				
N	99	429		
Low birthweight (<2500g)	8 (8.1%)	8 (1.9%)	3.80 (1.41-10.21)	4.04 (1.37-11.87)
Normal birthweight (2500-<4000g)	75 (75.8%)	315 (73.4%)	1.00	1.00
High birthweight (≥4000)	16 (16.2%)	106 (24.7%)	0.65 (0.36-1.16)	0.64 (0.34-1.21)
**Gestational age**				
N	100	432		
Preterm (≤36 weeks)	8 (8.0%)	15 (3.5%)	2.20 (0.93-5.21)	2.15 (0.85-5.44)
Term (37-41 weeks)	87 (87.0%)	384 (88.9%)	1.00	1.00

© 2024 Ebrahimi F et al. *JAMA Network Open*.

Post-term (≥42 weeks)	5 (5.0%)	33 (7.6%)	0.66 (0.25-1.76)	0.61 (0.22-1.69)
**Birthweight for gestational age**				
N	99	429		
SGA (<10th percentile)	19 (19.2%)	34 (7.9%)	2.68 (1.42-5.08)	2.70 (1.33-5.50)
AGA (10-90th percentile) (reference)	75 (75.8%)	355 (82.8%)	1.00	1.00
LGA (>90th percentile)	5 (5.1%)	40 (9.3%)	0.54 (0.21-1.42)	0.47 (0.17-1.35)

* Model I: Conditional logistic regression matched for age at index date, sex, calendar year, and county of residence.
**Model II: Model I and further adjusted for maternal age, maternal early-pregnancy body mass index, maternal country of birth, parity, highest level of education in parents, and smoking in early pregnancy.
Abbreviations: AGA, appropriate for gestational age; CI, Confidence interval; LGA, large for gestational age; MASLD, metabolic dysfunction-associated steatotic liver disease; aOR, adjusted odds ratio; SGA, small for gestational age.

© 2024 Ebrahimi F et al. *JAMA Network Open*.

eTable 8. Characteristics of MASLD Individuals and their Siblings at Birth and at Index date

Characteristic	MASLD (n=108)	Siblings (n=156)	*p* value
**Characteristics at birth**			
Maternal age (years)			
Median (IQR)	28.3 (25.6-32.5)	28.9 (25.3-32.7)	0.77
Age categories, n (%)			
≤24	20 (20.8%)	30 (23.4%)	0.93
25-29	35 (36.5%)	43 (33.6%)	
30-34	27 (28.1%)	34 (26.6%)	
≥35	14 (14.6%)	21 (16.4%)	
Birth year, n (%)			
1992-1999	58 (60.4%)	87 (68.0%)	0.21
2000-2010	37 (38.5%)	37 (28.9%)	
2011-2016	1 (1.0%)	4 (3.1%)	
Maternal country of birth, n (%)			
Nordic country	75 (78.1%)	94 (73.4%)	0.42
Other	21 (21.9%)	34 (26.6%)	
Living with partner			
Yes	88 (91.7%)	118 (92.2%)	0.89
No/missing	8 (8.3%)	10 (7.8%)	
Maternal smoking in early pregnancy			
Non-smoking	70 (72.9%)	94 (73.4%)	0.09
1-9 cig/day	8 (8.3%)	20 (15.6%)	
≥10 cig/day	10 (10.4%)	11 (8.6%)	
Missing	8 (8.3%)	3 (2.3%)	
Parity			
0	28 (29.2%)	43 (33.6%)	0.13
1	44 (45.8%)	42 (32.8%)	
≥2	24 (25.0%)	43 (33.6%)	
Maternal BMI at first visit (kg/m2)			
Median (IQR)	25.3 (23.0-29.9)	25.3 (23.1-30.3)	0.91
Range, min-max	16.5; 37.0	17.9; 40.8	
BMI Categories, n (%)			
<18.5	1 (1.0%)	3 (2.3%)	0.83
18.5 - <25	36 (37.5%)	50 (39.1%)	
25 - <30	23 (24.0%)	30 (23.4%)	
≥30	20 (20.8%)	29 (22.7%)	
Missing	16 (16.7%)	16 (12.5%)	
Birthweight (gram), n (%)			
Median (IQR)	3405 (3105-3773)	3423 (3140-3810)	0.77
Range, min-max	1160-4515	568-4840	
Categories, n (%)			
Low birthweight (1500-<2500g)	8 (8.3%)	9 (7.1%)	0.44
Normal birthweight (2500-<4000g)	76 (79.2%)	95 (74.2%)	
High birthweight (≥4000g)	12 (12.5%)	24 (18.8%)	
Missing	0	0	
Gestational age at birth (weeks), n (%)			
Median (IQR)	39.9 (39.0-40.6)	40.0 (38.9-40.8)	0.65
Range, min-max	30.9-42.7	23.1-43.1	
Categories, n (%)			
Preterm (<37 weeks)	7 (7.3%)	9 (7.0%)	0.94
Full term (37-41 weeks)	86 (89.6%)	111 (86.7%)	0.51
Post term (≥42 weeks)	3 (3.1%)	8 (6.3%)	0.28
Birthweight for gestational age			
SGA (<10th percentile)	16 (16.7%)	16 (12.5%)	0.68
AGA (10-90th percentile)	73 (76.0%)	102 (79.7%)	
LGA (>90th percentile)	7 (7.3%)	10 (7.8%)	
Missing	0	0	
Mode of delivery, n (%)			
Vaginal non-instrumental delivery	86 (89.6%)	110 (85.9%)	0.06
Vaginal instrumental delivery	0	7 (5.5%)	

© 2024 Ebrahimi F et al. *JAMA Network Open*.

Caesarean section	10 (10.4%)	11 (8.6%)	
Maternal complications			
Pre-gestational diabetes, n (%)	1 (1.0%)	0	0.25
Gestational diabetes, n (%)	2 (2.1%)	6 (4.7%)	0.30
Pre-eclampsia, n (%)	12 (12.5%)	15 (11.7%)	0.86
**Characteristics in study subjects at date of MASLD diagnosis/follow-up**			
Sex, n (%)			
Female	37 (38.5%)	68 (53.1%)	0.03
Male	59 (61.5%)	60 (46.9%)	
Age at diagnosis/index date (years)			
Median (IQR)	12.5 (7.6-16.3)	12.3 (6.5-16.9)	0.70
Age categories, n (%)			
Children (≤10y)	37 (38.5%)	57 (44.5%)	
Adolescents (11-17y)	46 (47.9%)	48 (37.5%)	0.28
Young adults (18-25y)	13 (13.5%)	23 (18.0%)	
Year at diagnosis/index date, n (%)			
1992-1999	7 (7.3%)	10 (7.8%)	
2000-2010	35 (36.5%)	46 (35.9%)	0.99
2011-2016	54 (56.3%)	72 (56.3%)	
Highest level of education in parents[#]			
≤9 years	6 (6.3%)	11 (8.6%)	
10 - 12 years	46 (47.9%)	57 (44.5%)	0.76
≥13 years	44 (45.8%)	60 (46.9%)	
MASLD histology, n (%)			
Simple steatosis	31 (32.3%)	–	–
MASH without fibrosis	12 (12.5%)	–	–
MASLD with non-cirrhotic fibrosis	51 (53.1%)	–	–
Cirrhosis	2 (2.1%)	–	–
Comorbidities, n (%)			
Cardiovascular Disease	7 (7.3%)	3 (2.3%)	0.08
Diabetes mellitus	6 (6.3%)	1 (0.8%)	0.02
Hypertension	2 (2.1%)	0	0.10
Dyslipidemia	0	1 (0.8%)	0.39

*Vaginal instrumental delivery refers to either vacuum extraction or forceps delivery.
[#]Education categories based on compulsory school, high school, and college.
Abbreviations: AGA, appropriate for gestational age; BMI, body mass index; LGA, large for gestational age; MASLD, metabolic dysfunction-associated steatotic liver disease; MASH, metabolic dysfunction-associated steatohepatitis; SGA, small for gestational age.

© 2024 Ebrahimi F et al. *JAMA Network Open*.

eTable 9. Birthweight, Gestational Age and Future Odds of MASLD – Sibling-Controlled Analysis

	MASLD	Siblings	aOR (95% CI)*	aOR (95% CI)**
**Birthweight**				
N	96	128		
Low birthweight (<2500g)	8 (8.3%)	9 (7.0%)	1.26 (0.36-4.33)	1.73 (0.46-6.55)
Normal birthweight (2500-<4000g) (reference)	76 (79.2%)	95 (74.2%)	1.00	1.00
High birthweight (≥4000g)	12 (12.5%)	24 (18.8%)	0.29 (0.10-0.84)	0.25 (0.08-0.79)
Birthweight as continuous variable by 100g increase	96	128	0.98 (0.93-1.04)	0.96 (0.91-1.02)
**Gestational age**				
N	96	128		
Preterm (≤36 weeks)	7 (7.3%)	9 (7.0%)	1.00 (0.28-3.59)	1.39 (0.35-5.49)
Full term (37-41 weeks) (reference)	86 (89.6%)	111 (86.7%)	1.00	1.00
Post-term (≥42 weeks)	3 (3.1%)	8 (6.3%)	0.29 (0.05-1.60)	0.23 (0.04-1.42)
**Birthweight for gestational age**				
N	96	128		
SGA (<10th percentile)	16 (16.7%)	16 (12.5%)	2.10 (0.78-5.62)	2.56 (0.90-7.30)
AGA (10-90th percentile) (reference)	73 (76.0%)	102 (79.7%)	1.00	1.00
LGA (>90th percentile)	7 (7.3%)	10 (7.8%)	1.08 (0.34-3.39)	1.39 (0.41-4.75)

Data are n (%) or aOR (95% CI).
* Model I: Conditional logistic regression conditioned on family and further adjusted for age, sex, and birthyear.
**Model II: Model I and further adjusted for maternal age, maternal early-pregnancy BMI, maternal country of birth, parity, highest level of education in parents, and smoking in early pregnancy.
Abbreviations: AGA, appropriate for gestational age; CI, Confidence interval; LGA, large for gestational age; MASLD, metabolic dysfunction-associated steatotic liver disease; aOR, adjusted odds ratio; SGA, small for gestational age.

© 2024 Ebrahimi F et al. *JAMA Network Open*.

eReferences

1. Fraser A, Ebrahim S, Smith GD, Lawlor DA. The associations between birthweight and adult markers of liver damage and function. Paediatric and perinatal epidemiology. 2008;22:12-21.
2. Sandboge S, Perälä M-M, Salonen MK, Blomstedt PA, Osmond C, Kajantie E, Barker DJP, et al. Early growth and non-alcoholic fatty liver disease in adulthood—the NAFLD liver fat score and equation applied on the Helsinki Birth Cohort Study. Annals of Medicine 2013;45:430-437.
3. Anderson EL, Howe LD, Fraser A, Callaway MP, Sattar N, Day C, Tilling K, et al. Weight trajectories through infancy and childhood and risk of non-alcoholic fatty liver disease in adolescence: The ALSPAC study. Journal of Hepatology 2014;61:626-632.
4. Breij LM, Kerkhof GF, Hokken-Koelega ACS. Risk for Nonalcoholic Fatty Liver Disease in Young Adults Born Preterm. Hormone Research in Paediatrics 2015;84:199-205.
5. Suomela E, Oikonen M, Pitkänen N, Ahola-Olli A, Virtanen J, Parkkola R, Jokinen E, et al. Childhood predictors of adult fatty liver. The Cardiovascular Risk in Young Finns Study. Journal of hepatology. 2016;65:784-790.
6. Bugianesi E, Bizzarri C, Rosso C, Mosca A, Panera N, Veraldi S, Dotta A, et al. Low Birthweight Increases the Likelihood of Severe Steatosis in Pediatric Non-Alcoholic Fatty Liver Disease. Official journal of the American College of Gastroenterology | ACG 2017;112:1277-1286.
7. Newton KP, Feldman HS, Chambers CD, Wilson L, Behling C, Clark JM, Molleston JP, et al. Low and High Birth Weights Are Risk Factors for Nonalcoholic Fatty Liver Disease in Children. J Pediatr 2017;187:141-146 e141.
8. Bedogni G, De Matteis G, Fabrizi M, Alisi A, Crudele A, Pizzolante F, Signore F, et al. Association of Bright Liver With the PNPLA3 I148M Gene Variant in 1-Year-Old Toddlers. J Clin Endocrinol Metab 2019;104:2163-2170.
9. Cantoral A, Montoya A, Luna-Villa L, Roldán-Valadez EA, Hernández-Ávila M, Kershenobich D, Perng W, et al. Overweight and obesity status from the prenatal period to adolescence and its association with non-alcoholic fatty liver disease in young adults: cohort study. BJOG : an international journal of obstetrics and gynaecology. 2020;127:1200-1209.
10. Amadou C, Nabi O, Serfaty L, Lacombe K, Boursier J, Mathurin P, Ribet C, et al. Association between birth weight, preterm birth, and nonalcoholic fatty liver disease in a community-based cohort. Hepatology 2022;76:1438-1451.
11. Thomas EL, Parkinson JR, Hyde MJ, Yap IK, Holmes E, Dore CJ, Bell JD, et al. Aberrant adiposity and ectopic lipid deposition characterize the adult phenotype of the preterm infant. Pediatr Res 2011;70:507-512.
12. Vasu V, Thomas EL, Durighel G, Hyde MJ, Bell JD, Modi N. Early nutritional determinants of intrahepatocellular lipid deposition in preterm infants at term age. Int J Obes (Lond) 2013;37:500-504.
13. Sipola-Leppanen M, Vaarasmaki M, Tikanmaki M, Matinolli HM, Miettola S, Hovi P, Wehkalampi K, et al. Cardiometabolic Risk Factors in Young Adults Who Were Born Preterm. American Journal of Epidemiology 2015;181:861-873.
14. Nobili V, Marcellini M, Marchesini G, Vanni E, Manco M, Villani A, Bugianesi E. Intrauterine growth retardation, insulin resistance, and nonalcoholic fatty liver disease in children. Diabetes care. 2007;30:2638-2640.
15. Faienza MF, Brunetti G, Ventura A, D'Aniello M, Pepe T, Giordano P, Monteduro M, et al. Nonalcoholic fatty liver disease in prepubertal children born small for gestational age: influence of rapid weight catch-up growth. Hormone research in pædiatrics : from developmental endocrinology to clinical research. 2013;79:103-109.
16. Malpique R, Bassols J, López-Bermejo A, Diaz M, Villarroya F, Pavia J, Congo A, et al. Liver volume and hepatic adiposity in childhood: relations to body growth and visceral fat. International Journal of Obesity 2018;42:65-71.
17. Erkamp JS, Jaddoe VWV, Mulders A, Steegers EAP, Reiss IKM, Duijts L, Gaillard R. Customized versus population birth weight charts for identification of newborns at risk of long-term adverse cardio-metabolic and respiratory outcomes: a population-based prospective cohort study. BMC Med 2019;17:186.

© 2024 Ebrahimi F et al. *JAMA Network Open*.

18. Mosca A, De Cosmi V, Parazzini F, Raponi M, Alisi A, Agostoni C, Nobili V. The Role of Genetic Predisposition, Programing During Fetal Life, Family Conditions, and Post-natal Diet in the Development of Pediatric Fatty Liver Disease. The journal of pediatrics. 2019;211:72-77.e74.

© 2024 Ebrahimi F et al. *JAMA Network Open*.
