# Supplementary material for: Birth Weight, Gestational Age, and Risk of Pediatric-Onset MASLD
Source: JAMA Netw Open. 2024 Sep 10;7(9):e2432420. doi: 10.1001/jamanetworkopen.2024.32420 (PMC11388034; doi:10.1001/jamanetworkopen.2024.32420)
Supplement: Supplement 2. — Data Sharing Statement [file jamanetwopen-e2432420-s002.pdf]

Data Sharing Statement

Ebrahimi. Birth Weight, Gestational Age, and Risk of Pediatric-Onset MASLD. *JAMA Netw Open*. Published September 10, 2024. doi:10.1001/jamanetworkopen.2024.32420

Data
Data available: No

Additional Information
Explanation for why data not available: Due to the confidentiality of data, the data which support the findings of this study are generally not available due to current regulations. However, requests for additional analyses might be considered upon reasonable request.
